# Development and Testing of a Data Capture Device for Use With Clinical Incentive Spirometers: Testing and Usability Study

**DOI:** 10.2196/46653

**Published:** 2023-09-07

**Authors:** Michael L Burns, Anik Sinha, Alexander Hoffmann, Zewen Wu, Tomas Medina Inchauste, Aaron Retsky, David Chesney, Sachin Kheterpal, Nirav Shah

**Affiliations:** 1 Department of Anesthesiology University of Michigan Health System Ann Arbor, MI United States; 2 Department of Electrical Engineering and Computer Science University of Michigan Ann Arbor, MI United States

**Keywords:** incentive, spirometry, Internet-of-Things, electronic health records, web-based intervention, medical device, medical tool, data collection, spirometry data, incentive spirometer, data analysis, algorithm, effectiveness

## Abstract

**Background:**

The incentive spirometer is a basic and common medical device from which electronic health care data cannot be directly collected. As a result, despite numerous studies investigating clinical use, there remains little consensus on optimal device use and sparse evidence supporting its intended benefits such as prevention of postoperative respiratory complications.

**Objective:**

The aim of the study is to develop and test an add-on hardware device for data capture of the incentive spirometer.

**Methods:**

An add-on device was designed, built, and tested using reflective optical sensors to identify the real-time location of the volume piston and flow bobbin of a common incentive spirometer. Investigators manually tested sensor level accuracies and triggering range calibrations using a digital flowmeter. A valid breath classification algorithm was created and tested to determine valid from invalid breath attempts. To assess real-time use, a video game was developed using the incentive spirometer and add-on device as a controller using the Apple iPad.

**Results:**

In user testing, sensor locations were captured at an accuracy of 99% (SD 1.4%) for volume and 100% accuracy for flow. Median and average volumes were within 7.5% (SD 6%) of target volume sensor levels, and maximum sensor triggering values seldom exceeded intended sensor levels, showing a good correlation to placement on 2 similar but distinct incentive spirometer designs. The breath classification algorithm displayed a 100% sensitivity and a 99% specificity on user testing, and the device operated as a video game controller in real time without noticeable interference or delay.

**Conclusions:**

An effective and reusable add-on device for the incentive spirometer was created to allow the collection of previously inaccessible incentive spirometer data and demonstrate Internet-of-Things use on a common hospital device. This design showed high sensor accuracies and the ability to use data in real-time applications, showing promise in the ability to capture currently inaccessible clinical data. Further use of this device could facilitate improved research into the incentive spirometer to improve adoption, incentivize adherence, and investigate the clinical effectiveness to help guide clinical care.

## Introduction

Pulmonary complications after major surgery, including pneumonia, atelectasis, respiratory failure, prolonged supplemental oxygen requirements, and reintubation, are common, expensive, and harmful to patients [1]. Studies estimated these complications in the range of 2%-39% [2,3], though atelectasis alone has been found to affect up to 92% of postsurgical patients [4]. Originating in the 1970s, the incentive spirometer was designed to mimic the physiology of a sigh or a yawn—a slow voluminous inhalation [5,6], and this basic medical device is often used in postoperative care to aid lung expansion to prevent or reduce respiratory complications.

To complicate the matter, correct use and device adherence is low among patients. Guidelines on how to properly use the incentive spirometer device outline best practices [7,8], but over 26% of patients fail to use their incentive spirometer correctly, and over 38% deny ever using their device in their postoperative care [9], highlighting the need for more evidence-based recommendations. There is sparse evidence for the use of the incentive spirometer device [10] for postoperative pulmonary complication prevention [11] with only a few studies demonstrating clinical effectiveness when used properly [12,13]. As a result of the lack of high-level evidence, some clinical practice guidelines do not support its routine postoperative use [14]. Additionally, there remains disagreement as to the most effective way to use the device, as studies have been unable to demonstrate its superiority over other techniques such as deep breathing techniques, directed coughing, early mobilization, and optimal analgesia [15]. Uncertainty around the effective spirometry use is partially due to the scarcity of spirometer compliance data [16]. Compliance measurements, made through self-reporting and staff observation, are difficult to obtain, and when captured, they have demonstrated low patient adherence to the incentive spirometer device [12]. Though data remain elusive, 86% of health care providers believe patient adherence is poor, and 95.4% believe it should be improved [17], demonstrating the perceived use of incentive spirometry. The first step in determining the optimal use of the incentive spirometer is to improve data collection. Automated capture of spirometry data may improve the quality of research studies and ultimately determine the incentive spirometer’s use in improving lung function and minimizing postoperative complications.

Incentive spirometers are simple plastic meters to measure inhalational breath flow and volume; they lack the ability to record data. Digital flowmeters can be used to replicate the incentive spirometer [18]. While an option for improving data capture, the digital flowmeter can be clinically infeasible in most practices due to its complexity and cost. Collecting data directly from incentive spirometers falls into a technology category called the “Internet-of-Things” [19]. There have been great advances in the miniaturization of computing devices and the evolution of the “Internet-of-Things” into mainstream health devices [20,21]. As a result, wearable technologies and web-based platforms are capturing more clinical data now than ever before [22,23]. These technologies give clinicians and researchers access to otherwise inaccessible patient data and the ability to investigate new data interactions, such as enabling patients to exercise with wearable device hardware (eg, chest monitors and watches) and incorporating and communicating these data with patient physiologic and movement data [24,25]. The goal was to use this technology for the incentive spirometer.

We hypothesize an add-on device can accurately measure flow and volume data from a common incentive spirometer. This paper describes the creation and testing of an incentive spirometer add-on device to measure flow and volume data. An Internet-of-Things approach was adopted to enable this device to work with an existing incentive spirometer to capture physiological data and communicate externally on a closed private internet connection using hospital Wi-Fi and Bluetooth technologies. Captured data were tested for use by developing a classification algorithm to determine valid from invalid breaths.

## Methods

### Ethics Approval

Institutional review board approval was obtained for this prospective exploratory study on April 15, 2021 (HUM00196543, University of Michigan, Ann Arbor). Informed consent was waived as participants were limited to authors within this study, and interventions for this study are limited to existing approved uses of the incentive spirometer.

### Add-On Device Creation

**Figure 1:**
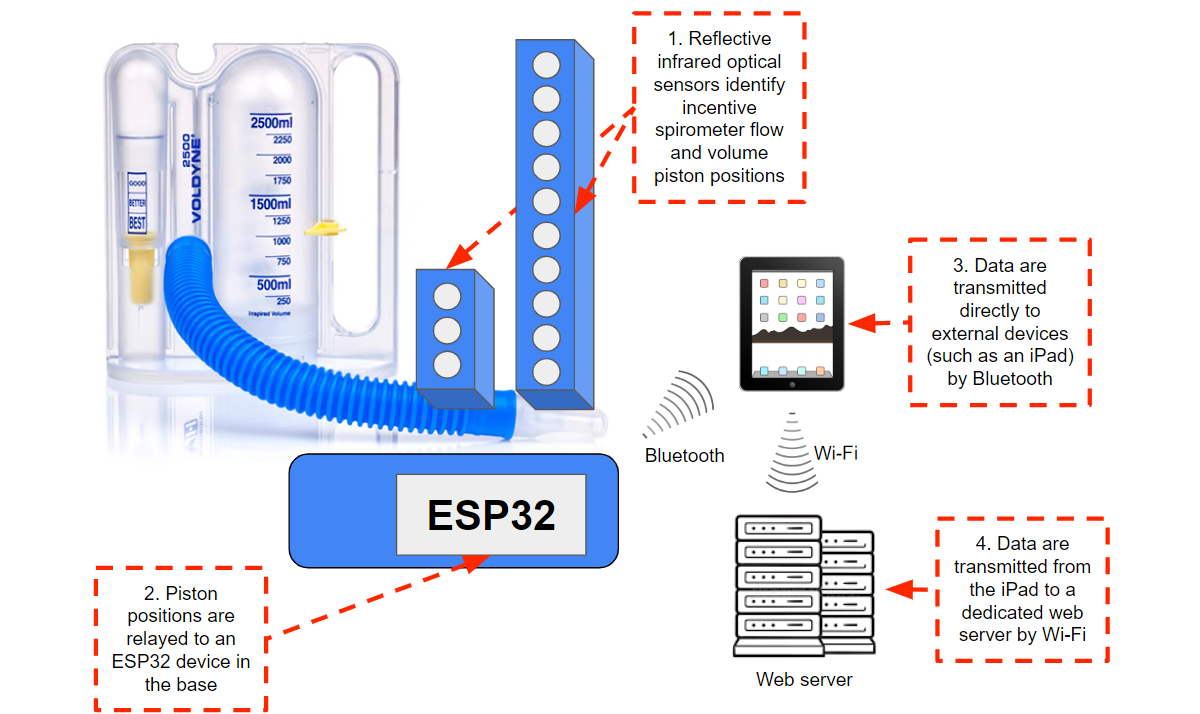
The incentive spirometer piston positions were read using reflective infrared optical sensors (1) and relayed to an ESP32 in the base of the add-on device (2). The device both stored the data internally and used Bluetooth technology to transmit spirometer data to an Apple iPad (3). Data were then transmitted from the iPad to dedicated servers for further data processing and storage (4).

**Figure 2:**
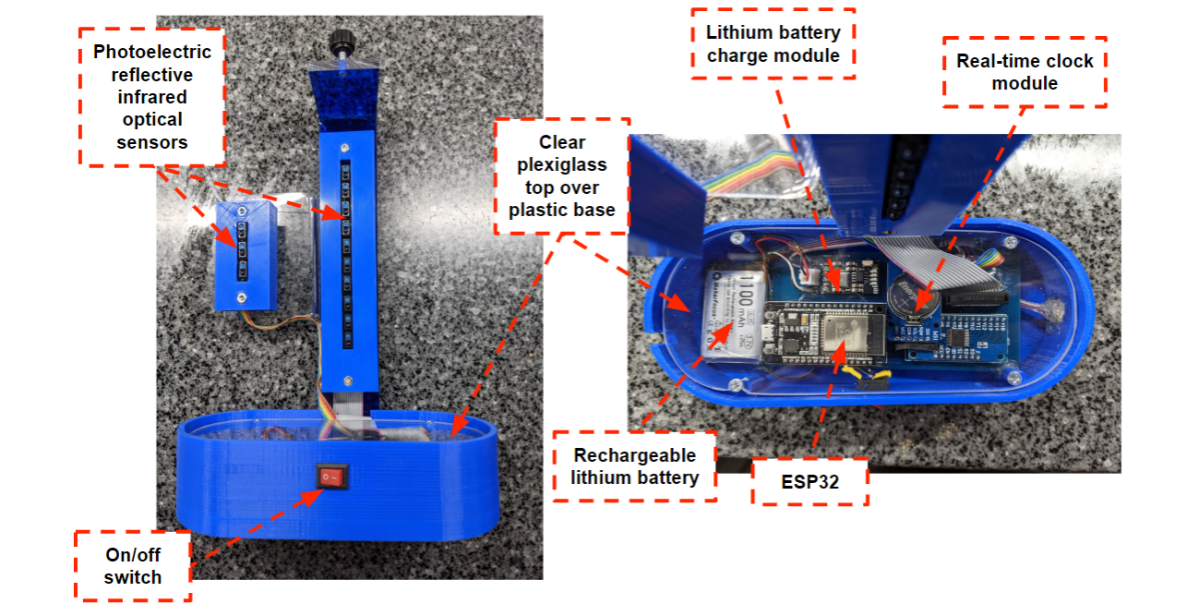
Ten photoelectric reflective infrared optical sensors were placed along the spirometer volume column with each sensor along a 500 mL marking on the incentive spirometer device (500-5000 mL); 3 sensors placed along the flow column. An on/off switch is featured on the front of the device. An ESP32 development board, rechargeable lithium battery, and real-time clock module are labeled. Connections between components were made with breadboard jumper wires and resistors, and all components are encased in a 3D-printed base with a layer of plexiglass overtop. A microsecure digital card reader module is in the back of the base (not shown).

**Figure 3:**
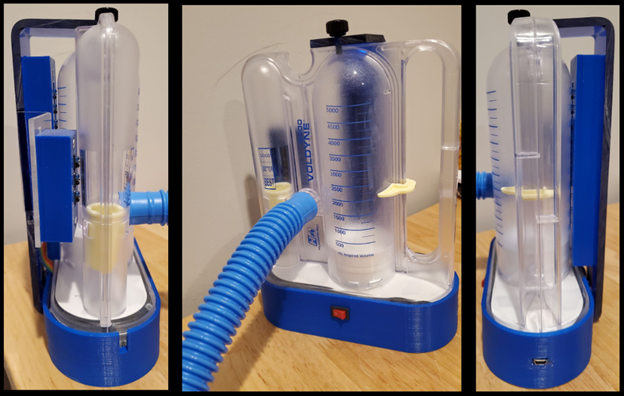
The Hudson RCI Voldyne 5000 Volumetric Exerciser (Teleflex Medical) incentive spirometer is situated over the base and locked into place with an overlying base lip and a screw on top of the 3D-printed posterior arm. A charging port is located in the base (right picture).

An add-on device was designed and created for use with the Hudson RCI Voldyne 5000 Volumetric Exerciser (Teleflex Medical) incentive spirometer (Figures 1-3 and Multimedia Appendix 1) and composed of photoelectric reflective infrared optical sensors (Xingyheng) positioned lateral to the spirometer volume and flow columns. Ten sensors were placed along the spirometer volume column with each sensor along a 500 mL marking on the incentive spirometer device (500-5000 mL). Three sensors were placed along the flow column corresponding to the middle of flow spirometer markings (“best,” “better,” and “good”). Sensors were connected to a microcontroller by a solderless breadboard, using an ESP32 development board (Dorhea), a low-cost low-power system-on-a-chip microcontroller with integrated Wi-Fi and dual-mode Bluetooth wireless communication capability. Components were soldered on a breadboard with breadboard jumper wires and resistors connecting components. Interactions between the microcontroller and the sensors were coded using C++ (Bell Laboratories of American Telephone and Telegraph). Components were encased in a 3D-printed base situated beneath the incentive spirometer with a layer of plexiglass fixed overtop.

Data were sent directly to an iOS application running on an iPad Pro (12.9″ and 10.2″, fifth generation, Apple) through ESP32 Bluetooth as well as stored on the add-on device in a 32 gigabyte microsecure digital card (Kootion) and card reader module (HiLetGo). A clock module (Melife) time-stamped data. Further interconnection was made possible using an analog digital multiplexer breakout board (Xie QianJin) and included a 3.7-V 2400-mAh rechargeable lithium battery (Akzytue) and charging module (MakerFocus) to power the device. An alternative design for the add-on base can be found in Multimedia Appendix 2.

### Software and Video Game Creation

An iOS video game and analytics application was built using Unity and C# (Microsoft). A web server receives the data and handles application programming interface (API) requests written in Java (Sun Microsystems). The ESP32 reads the sensor data using custom C++ code and sends processed data to an Apache web server via Wi-Fi through an iPad device connected via Bluetooth (Figure 1). Python (Python Software Foundation) applications were exposed by the web server, and downstream applications were networked with the server through an API to allow data collection and further use. A web server receives the data and handles API requests. A video game was developed specifically for use with the incentive spirometer and add-on device serving as a controller, designed for use with an Apple iPad Pro.

### Add-On Device Sensor Testing

Two investigators (AS and MLB) tested the add-on device without crossover using the Hudson RCI Voldyne 5000 Volumetric Exerciser incentive spirometer. In these tests, 5 user breaths were attempted at each of the 10 volume sensor positions (100-5000 mL) for a total of 50 breaths per user. The volume goal was to get the top of the volume piston to the desired volume marking, while the flow goal was to get the top of the flow bobbin to the middle of the desired flow marking. Flow readings were also tested with every volume test with an additional 5 flow tests at each of the 3 flow sensor positions. To attain the desired flow or volume piston levels, users were allowed to breathe through or tilt the incentive spirometer device.

A single investigator (MLB) tested flow and volume sensor ranges with the incentive spirometer connected to the Puritan-Bennet PTS-2000 Ventilator Analyzer Tester (Mallinckrodt) digital flowmeter. In this testing, the investigator attempted increasing volume and flow values to identify sensor ranges. Volume measurements from the flowmeter were corrected using the body temperature, pressure, water vapor saturated method (correcting for body temperature [37 °C], ambient pressure, and gas saturated with water vapor). The flowmeter volume was calculated to be 183.75 mL by calibrating the flowmeter readings precisely at the 1000 mL display level of the Voldyne 5000, averaged across 10 breaths. This volume was subtracted from each raw volume reading from the flowmeter. Flowmeter testing was attempted with a minimum of 25 breaths around each flow and volume sensor level, independently, and the volume achieved from the flowmeter and the sensor level attained from the iPad were recorded. Due to limitations continually achieving breath volumes above 3250 mL (sensor level 7), additional testing using the 2500 mL volume Hudson RCI Voldyne 2500 Volumetric Exerciser (Teleflex Medical) incentive spirometer was conducted. The add-on device was created with each sensor aligning to the 500 mL markings of the Voldyne 5000. When using the Voldyne 2500, the sensors were near but incompletely aligned to the 250-mL markings.

### Breath Algorithm Development and Testing

While sensors in the add-on device can detect where a piston is located, they cannot determine if a breath was conducted to achieve the sensor level. It is important to be able to accurately identify when a breath moves the incentive spirometer piston and bobbin as opposed to tilting the incentive spirometer causing them to fall to the desired level. To aid in distinguishing a breath attempt, an algorithm was developed in Python (version 3.1) to process and classify each user's breath using flow and volume data over time. This algorithm reads spirometer log data into a pandas data frame and parses data by identifying the start and end of breaths. Breath start was identified by a zero flow value that precedes a positive flow value, while breath end is identified by 2 consecutive zero flow values. Once identified, each breath is classified as “valid” or “invalid.” A valid breath must have the following volume criteria: volume starts at 0, increases within 1.5 seconds of breath start, >0 throughout the breath, does not decrease while there is positive flow, and the length of the breath is between 2 and 15 seconds. Breaths that do not meet these requirements were deemed invalid.

To test this algorithm, separate from device testing, 2 investigators (AS and MLB) each attempted to create 5 valid and 5 invalid breaths at each volume sensor level. These breath data were processed through the classification algorithm and evaluated. Invalid breaths were created by manually tipping the incentive spirometers or starting breaths at a starting position >0. All data validation was retrospectively validated using expert opinion from one of 2 users (AS and MLB).

### Statistical Analysis

Summary and descriptive statistics were calculated for collected data using basic statistical techniques to assess models created for breath analysis including accuracy (as defined by user identification as gold standard), as well as mean, median, and SD for volume and flow measurements in sensor range testing.

## Results

### Add-On Device Sensor Testing

An add-on incentive spirometer device was created to independently measure real-time spirometer flow and volume piston positions (Figures 1-3). From 2 investigators, 99 of 100 volume readings were measured at the correct corresponding volume sensor (accuracy 99%, SD 1.4%). Only a single volume reading was inaccurate, failing once to capture at the lowest volume sensor (500 mL). All 130 flow bobbin readings corresponded to the correct flow sensor (100% accuracy).

**Figure 4:**
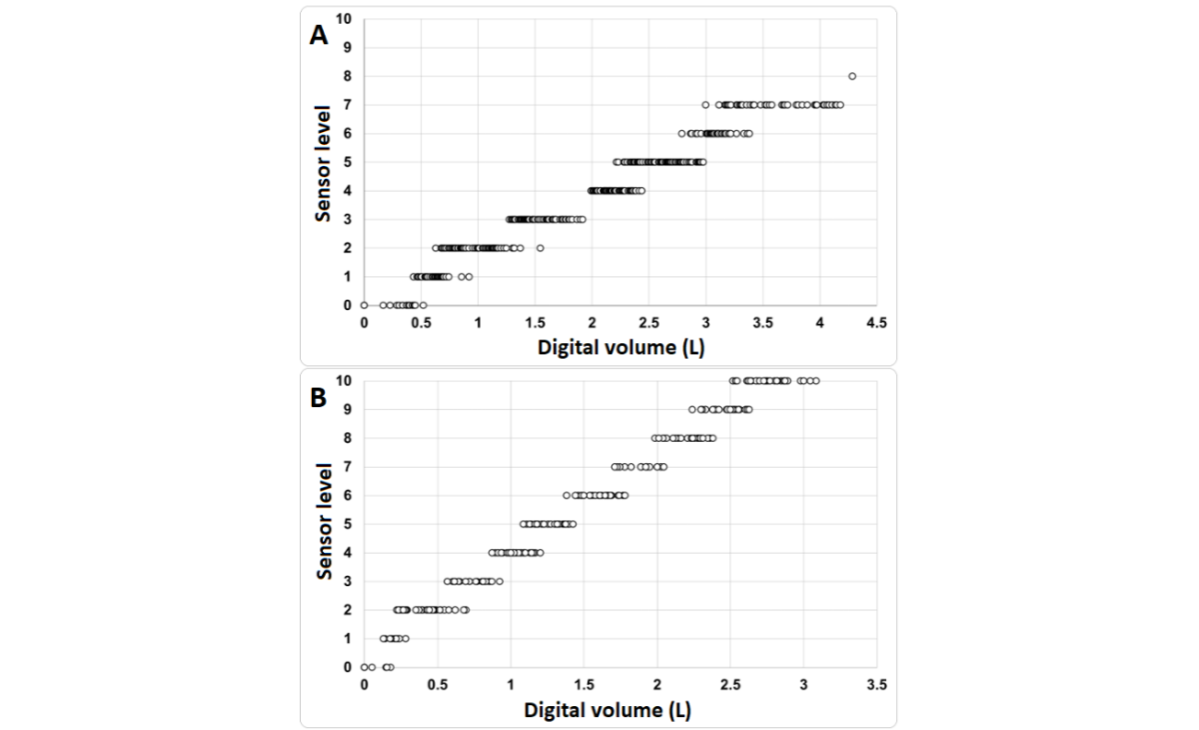
User testing of add-on device connected to a digital flowmeter for both the Voldyne 5000 (A) and the Voldyne 2500 (B) incentive spirometers. Circles represent individual breath attempts with digital flowmeter volume readings (L, x-axis) and add-on device sensor iPad readings (y-axis). Sensor levels were designed to be placed at 500 mL levels of the Voldyne 5000 incentive spirometer and closely aligned to 250 mL markings of the Voldyne 2500.

Volume and flow sensor ranges were determined using a digital flowmeter in line with the 5000 and 2500 mL incentive spirometers independently (Figure 4 and Tables 1 and 2). Add-on sensors were designed with placement at every 500 mL marking on the Voldyne 5000 incentive spirometer. These sensor placements were not adjusted for the 2500 mL incentive spirometer testing. Investigator breath limitations were reached at volume levels approaching 4000 mL. Between the 2 devices tested, median and average volumes were within 7.5% (SD 6%) of target volume sensor levels. Maximum triggering values seldom exceeded the intended sensor level, showing a good correlation to placement. Flow levels corresponding to “best,” “better,” and “good” levels differed significantly between devices with the 5000 mL flow values more than double the 2500-mL incentive spirometer at each level.

**Table 1:** Volume sensor ranges.^a^

Number		Breaths, n	Volume (L)			
			Mean (SD)	Median (IQR)	Minimum	Maximum
**Voldyne 5000 volume sensor**						
	1	43	0.61 (0.10)	0.62 (0.54-0.67)	0.43	0.92
	2	71	0.98 (0.20)	0.99 (0.81-1.13)	0.63	1.55
	3	74	1.55 (0.18)	1.54 (1.40-1.68)	1.27	1.92
	4	60	2.20 (0.12)	2.17 (2.11-2.28)	1.99	2.44
	5	88	2.59 (0.20)	2.59 (2.42-2.76)	2.21	2.98
	6	37	3.09 (0.13)	3.10 (3.02-3.16)	2.79	3.38
	7	48	3.59 (0.35)	3.53 (3.29-3.96)	3.00	4.18
	8	1	4.29 (N/A^b^)	4.29 (N/A)	4.29	4.29
	9	0	N/A (N/A)	N/A (N/A)	N/A	N/A
	10	0	N/A (N/A)	N/A (N/A)	N/A	N/A
**Voldyne 2500 volume sensor**						
	1	13	0.21 (0.04)	0.22 (0.18-0.23)	0.13	0.28
	2	35	0.41 (0.13)	0.43 (0.29-0.48)	0.22	0.69
	3	21	0.73 (0.11)	0.72 (0.64-0.82)	0.57	0.92
	4	29	1.03 (0.09)	1.02 (0.99-1.09)	0.87	1.20
	5	23	1.26 (0.10)	1.27 (1.18-1.34)	1.09	1.43
	6	24	1.60 (0.11)	1.61 (1.53-1.67)	1.38	1.78
	7	16	1.89 (0.11)	1.91 (1.81-1.96)	1.71	2.05
	8	25	2.21 (0.11)	2.24 (2.13-2.28)	1.99	2.38
	9	26	2.47 (0.11)	2.49 (2.40-2.55)	2.24	2.63
	10	37	2.75 (0.14)	2.74 (2.64-2.85)	2.52	3.09

^a^Results from investigations of increasing volume sensor readings from the Voldyne 2500 and Voldyne 5000 incentive spirometers. The number of breaths (n) at each sensor level (1-10) and the average, median (IQR), minimum, and maximum readings were obtained from a digital flowmeter in liters (L).

^b^N/A: not applicable.

**Table 2:** Flow sensor ranges.^a^

		Breaths, n	Flow (L/minute)			
			Mean (SD)	Median (IQR)	Minimum	Maximum
**Voldyne 5000 flow sensor**						
	Best	30	26.36 (5.56)	26.04 (22.51-27.76)	19.21	40.21
	Better	30	44.31 (5.49)	43.56 (40.44-48.25)	36.10	56.69
	Good	31	57.75 (7.74)	58.24 (53.99-61.27)	39.36	71.38
**Voldyne 2500 flow sensor**						
	Best	30	12.24 (2.36)	12.31 (10.69-13.12)	8.43	19.63
	Better	30	18.70 (1.92)	19.08 (17.45-19.56)	15.07	22.60
	Good	31	28.45 (3.34)	28.13 (25.93-29.96)	22.61	38.30

^a^Results from investigations of increasing flow sensor readings from the Voldyne 2500 and Voldyne 5000 incentive spirometers. The number of breaths (n) at each sensor level (“best,” “better,” and “good”) and the average, median (IQR), minimum, and maximum readings were obtained from a digital flowmeter in liters per minute (L/minute).

### Breath Algorithm Development and Testing

**Figure 5:**
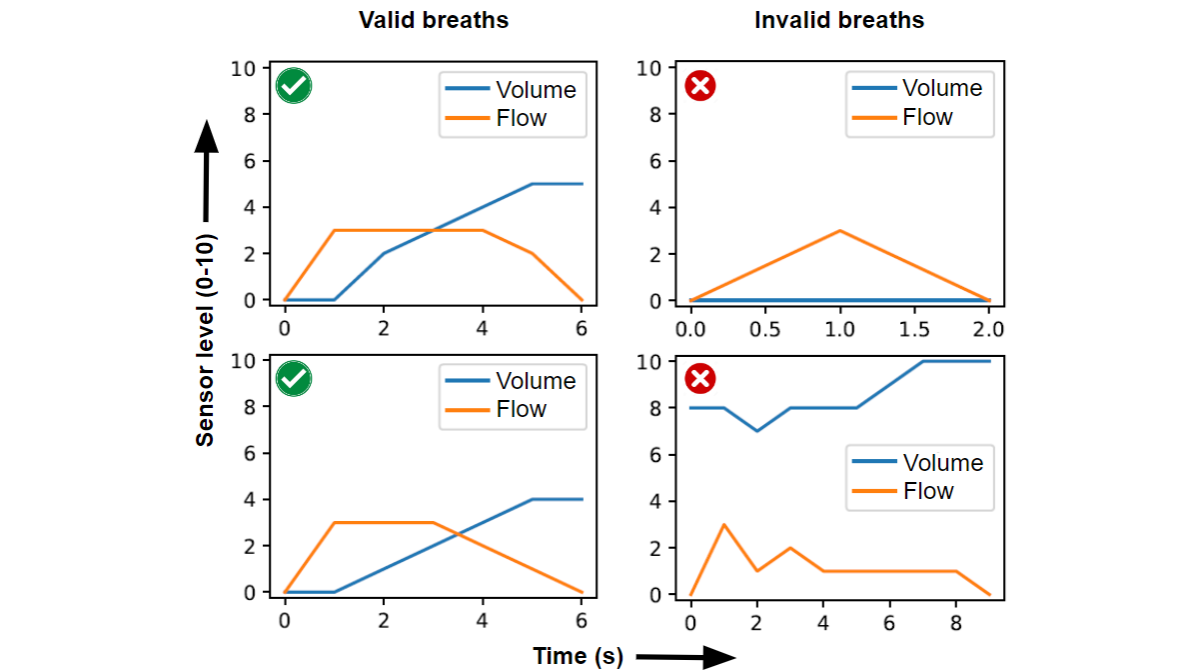
Example results from the breath algorithm used to classify “valid” from “invalid” breath attempts. The y-axis represents sensor levels for flow and volume readings in the incentive spirometer device: flow (0-3; corresponding to none: 0; “good”: 1; “better”: 2; and “best”: 3); volume (0-10, representing each 500 mL increment from 0 to 5000 mL). The x-axis represents the time from breath start in seconds.

After data collection, an algorithm was developed to classify breath data to determine when the spirometer was actively used. This algorithm works by classifying each individual breath using criteria to determine their validity as described in the Methods section. Independent of sensor testing, 2 members of the study team attempted 5 valid and 5 invalid breaths at each of ten 500-mL volume levels (500-5000 mL). Investigator limitations in achievable volumes resulted in a total of 65 valid breaths and 100 invalid breaths. The breath classification algorithm resulted in a 100% sensitivity and a 99% specificity for the classification of “valid” versus “invalid” user breaths. A single valid reading was inappropriately classified by the algorithm, occurring at the 500 mL volume sensor level. Example breath algorithm results are shown in Figure 5.

**Figure 6:**
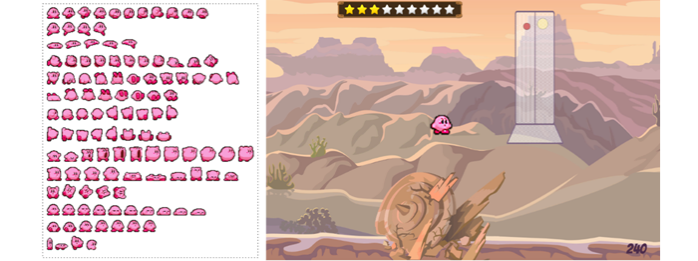
A video game was specifically designed for use with the incentive spirometer and add-on device based on the Kirby Nintendo character. A sprite sheet for the character development within the video game is shown on the left with a screenshot of the video game shown on the right. Multimedia Appendix 3 shows the game being controlled using an incentive spirometer.

To investigate the downstream applicability of breath data, a video game was developed using Unity software, Apple iOS, with a single final application installed on Apple iPad devices (Figure 6). The game was a Kirby (Nintendo)-based side scroller, where the character would approach an obstacle and traverse the obstacle after a successful breath. This game was developed specifically for use with the incentive spirometry device with game-play centered around proper use [7,8]. The incentive spirometer with an add-on device was successfully used to control the created game. A video showing the game played in real time with an incentive spirometer device can be found in Multimedia Appendix 3.

## Discussion

### Principle Findings

In this study, an add-on device was created to allow the capture of incentive spirometry data with high accuracy (99%, SD 1.4% volume and 100% flow when tested at incentive spirometer level markings). Maximum triggering values were rarely exceeding intended sensor levels, proving excellent differentiation of levels in the design. Furthermore, while sensor placements were designed around the Voldyne 5000 incentive spirometer, the similarly shaped Voldyne 2500 performed well in testing without sensor modifications, suggesting use of the add-on device to similar incentive spirometers without significant redesign. While range testing was not intended to study volume sensor accuracy, between the 2 spirometers tested, median and average volumes were within 7.5% (SD 6%) of target volume levels, with the worst individual readings at lower volumes (250-500 mL sensors) with readings maximally 40 to 120 mL off target, respectively.

To differentiate quality breath attempts from errors, a classification breath algorithm was developed. The device allowed identification of start and end of breath attempts, valid from invalid breaths assessment, maximum flow, maximum volume, and volume or time ascents and descent calculations. The breath classification algorithm used device data to discern valid versus invalid breath attempts, showing 100% sensitivity and 99% specificity. Identifying valid breath data from noise is critically important for downstream applications and, while the classification algorithm yielded results, further efforts for improvement could be made using additional rule-based systems or machine learning algorithms.

This system is intended to enable clinical providers access to previously inaccessible spirometry data to improve spirometer instruction and use protocols, study patient compliance, and incentivize use. Using an add-on device similar to what was created in this study would increase the granularity of spirometer compliance data and could be used to provide insight into proper incentive spirometer use. Additionally, the add-on device can allow focused interventions to improve adherence. Reminder notifications alone have been shown to improve incentive spirometer use. In one study, an add-on use-tracking device was equipped with a bell that sounded for up to 2 minutes every hour as a reminder for the patient to use their incentive spirometer [12]. This study demonstrated that patients using the reminder device had a greater number of mean daily inspiratory breaths and a percentage of recorded hours with an inspiratory breath event. More importantly, patients with the reminder displayed significantly lower mean atelectasis severity scores measured by chest radiography, reduced median postoperative and intensive care unit length of stay, and had a lower mortality rate at 6 months. These findings support postoperative incentive spirometer use and show effectiveness of a simple intervention to improve incentive spirometer adherence.

### Gamification

To demonstrate the real-time use of incentive spirometry data, iPad video game was created to be controlled by the add-on incentive spirometer device. In testing, the game showed no appreciable lag and continued connectivity during use, proving electronic spirometer data collected by the add-on device to be capable of real-time gamification applications. Gamification of medical interventions is an exciting concept for improving medical care adherence. Breathing games for the incentive spirometer is a familiar idea, with one group brainstorming a suite of games for asthmatics focusing on breathing metaphors as incentives for spirometer use [26], while others developed video games to incentivize breathing exercises and peak expiratory flow using digital flowmeters [18,27]. There exists an abandoned patent around the use of the incentive spirometer as a game controller [28] and an active patent around use of the flowmeter in video games [29], further supporting the popularity of the idea. The device in this study was created leveraging recent technologies and focusing design on clinical care use. Using the add-on device, as opposed to a digital flowmeter, maintains the current use of incentive spirometers in medical settings to allow native data capture. Potential reuse of the add-on device limits additional costs such as those incurred using digital flowmeters.

### Limitations

Limitations to the add-on device design include contamination risks, costs, and technical and workflow implementation constraints. First, while completely enclosed, the add-on device is designed to be reused and carries the risk of infection—an especially important consideration in a respiratory pandemic such as COVID-19. To improve sterility, the device was enclosed in plastic, and sensors were placed behind the incentive spirometer, removing the need to expose the base to breaths from sensors placed below. Improvements can be made to close remaining gaps in the plastic encasing to further enclose the device and allow cleaning like an iPad or PlayStation controller, commonly used in the hospital settings. Second, routine incentive spirometer postoperative care has been estimated to carry a US $107.36 cost per patient above material cost of the spirometer device, which totals US $1.04 billion in total US annual costs [30]. This is a significant cost for a device with sparse evidence around use and poor patient compliance. While incentive spirometer devices are not reusable from patient to patient, the add-on device was designed to be reusable, lowering its effective cost. Overall, the add-on device carries a material price of approximately US $150 per unit (Multimedia Appendix 4), which could be reduced by bulk purchasing and further investigation into alternative individual components. While add-on device cost is an addition to the already significant price, identifying compliance and improving adherence will facilitate improved use and function of the incentive spirometer. Further studies of the incentive spirometer are required to investigate the prevention of postoperative breathing complications and their associated health care costs. These studies are dependent upon accurate compliance data and would benefit from the capabilities of the add-on device. Third, there exist integration and maintenance requirements of the add-on device, and it may be feasible for use only in hospital systems with existing technical support structures. The device was designed to minimize technical requirements, but more investigation is required. Future studies are required to trial the add-on device in clinical settings and test for improved adherence using strategies such as gamification compared to traditional incentive spirometry.

### Conclusions

Incentive spirometers are routinely used in hospital settings, specifically in postoperative clinical care, but recommendations for proper routine use lack thorough investigation due to a general lack of data on device use. Creating a low-cost, effective, and reusable add-on device for the incentive spirometer allows native collection of previously inaccessible incentive spirometer compliance data. These data can facilitate research into incentive spirometer use to guide clinical care, incentivize adherence, and draw conclusions about the clinical effectiveness of the incentive spirometer.

## Appendix

Multimedia Appendix 1 Add-on device design and schematics.

Multimedia Appendix 2 Alternative design schematic for add-on device.

Multimedia Appendix 3 Video displaying an investigator (TM) using the incentive spirometer with the add-on device to control the created video game.

Multimedia Appendix 4 Add-on device cost.

## Abbreviations

API: application programming interface

## Data Availability

Deidentified data were collected from spirometer use by the study team. Data were collected using the incentive spirometer, add-on device, and digital flowmeter. Data sets generated and analyzed within this study are available from the corresponding author upon reasonable request.
