# Supplementary material for: Development and Testing of a Data Capture Device for Use With Clinical Incentive Spirometers: Testing and Usability Study
Source: JMIR Biomed Eng. 2023 Sep 7;8:e46653. doi: 10.2196/46653 (PMC11041496; doi:10.2196/46653)
Supplement: Multimedia Appendix 1 [file biomedeng_v8i1e46653_app1.docx]

**Multimedia Appendix 1. Add-on device design and schematics.**

Electronic schematics of the base and columns (flow and volume), showing electrical components and sensor placements can be found in Figure S1, while 3-dimensional renderings of the plastic components for the 3D-printed base and the flow columns for the add-on device can be found in Figure S2.

**
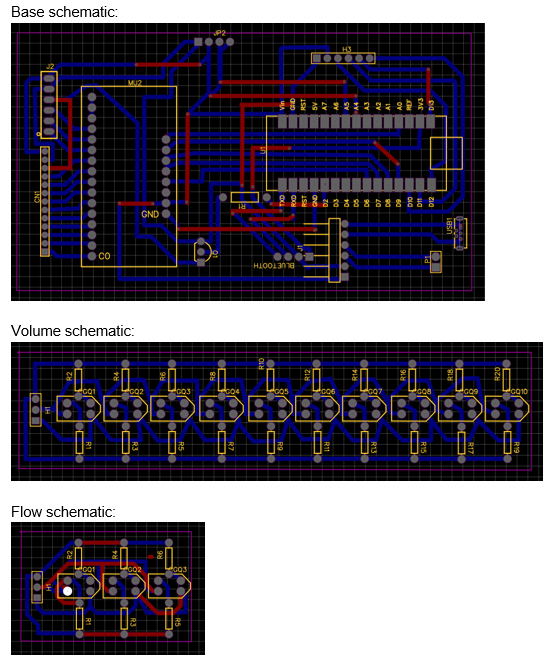
**

**Figure S1.** Electronic schematics of the base and columns (flow and volume), showing electrical components and sensor placements, labeled a, b, and c, respectively.


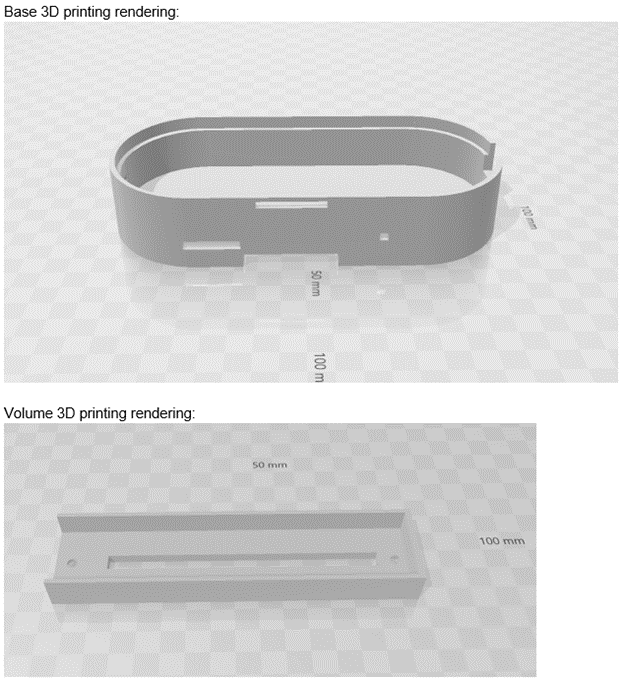


**Figure S2.** 3-dimensional renderings of the plastic components for the 3D-printed base and the flow column for the add-on device, labeled a and b, respectively.

Several improvements were made to original designs to decrease the size and cost of the add-on device and improve functionality. Critical improvements were made around sensor design and calibration. The add-on device in this study electronically collects incentive spirometer data using photoelectric reflective infrared optical sensors independently measuring real time spirometer flow and volume piston positions (Figures 1 and 2). By design, these sensors measure the presence of an object within 12mm of the receiver and by determining which sensors were triggered, we could tell where each piston was in their respective chamber at any time. An earlier design (Multimedia Appendix 2) positioned two distance sensors underneath the volume and flow sensors, but as the pistons moved farther away from the sensors, the sensors became less accurate, with the flow sensor capturing <50% accuracy in early testing. Changing from distance sensors below the incentive spirometer to a series of multiple detection sensors positioned behind the spirometer greatly improved accuracy and potential sterility of the device. User testing demonstrated near perfect sensor capture.

Moving sensors out of the base provided space to incorporate additional improvements. The final design incorporated a microSD card reader for internal data storage, allowing data collection independent of connection to an external application, an important consideration when attempting to capture all spirometer use data. We added a rechargeable lithium battery, charging module, and on/off switch to improve ease of use allowing the add-on device to be mobile and self-contained, free from wired connections. The add-on device captures native incentive spirometer compliance data and transmits over existing hospital networks using Wi-Fi and Bluetooth technologies. We transitioned from an Arduino Raspberry Pi configuration to the ESP32 (a low-cost, low-power system-on-a-chip microcontroller with integrated Wi-Fi and dual-mode Bluetooth), improving connectivity, and removing hardware bulk from the original design while maintaining data processing through a web server for use in downstream applications. Connectivity allows secure data passage over hospital Wi-Fi networks, avoiding open network communications, limiting data leakage, and allowing the potential for remote monitoring. The entire base is encased for sterility allowing cleaning and reuse, of specific importance in the COVID-era.

*Ambient Light and Sensor Calibrations*

Light from external sources, such as natural light, contain wavelengths outside of the visual spectrum which can throw off the sensors used in the add-on device. To reduce interference by direct ambient light and subsequent noise in sensor data, sensors were recalibrated at each breath within the Unity application. All add-on device testing was conducted indoors with ambient sunlight and room lighting. While there was no specific wavelength testing, the more direct sunlight exposure to the sensors the more calibration was required and the greater the inaccuracies. Because of this potential interference, sensors were recalibrated for each breath to allow for changing light conditions during use, such that if sensors were calibrated in the dark and then used near a window the raw sensor reading could adapt. This design allows the timing of calibration to change from a single calibration when the device was turned on to calibrations prior to every breath. In this method, a user can move with the device into or out of ambient light between breaths without appreciable interference, further improving capture accuracy and decreasing noise within the data.
