# Supplementary material for: Development and Testing of a Data Capture Device for Use With Clinical Incentive Spirometers: Testing and Usability Study
Source: JMIR Biomed Eng. 2023 Sep 7;8:e46653. doi: 10.2196/46653 (PMC11041496; doi:10.2196/46653)
Supplement: Multimedia Appendix 2 [file biomedeng_v8i1e46653_app2.docx]

**Multimedia Appendix 2. Alternative design schematic for add-on device.**

There were two major hardware designs for the add-on incentive spirometer device, based on the location of sensors. An original, alternative design had distance sensors located at the bottom of the incentive spirometer, below the volume and flow pistons, located within a mounted base (Figure S1). The final design, presented throughout the manuscript, had position sensors lateral to the volume and flow chambers mounted behind the incentive spirometer device. This change was made to improve device reading accuracy and decrease medical contamination of the add-on device as having sensors below the spirometer required holes to be punched in the bottom of the spirometer to function (Figure S2). To improve sterility, a layer of plexiglass was added to the base, but the plexiglass caused the distance sensors to read inconsistently, further necessitating the change in sensor design. Both designs utilized a 3D-printed base to sit beneath the incentive spirometer. In the original hardware design, the sensors themselves were different from the final design. The original design used an integration reflection type photoelectric detector with a gallium arsenide infrared diode transmitter and a silicon plane photoelectric triode receiver (Uxcell, ST188) to measure the distance of each of the pistons from the sensors. The final design used sensors to determine presence of an object as described in the manuscript.

**
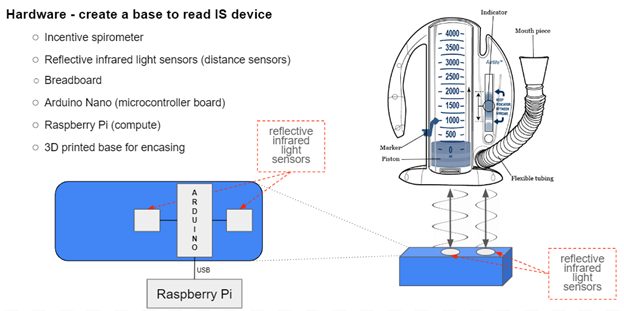
Figure S1.** Schematic of an alternative incentive spirometry add-on device design, with distance reflective light sensors located below the incentive spirometer, and connectivity via an Arduno Nano and Raspberry Pi.


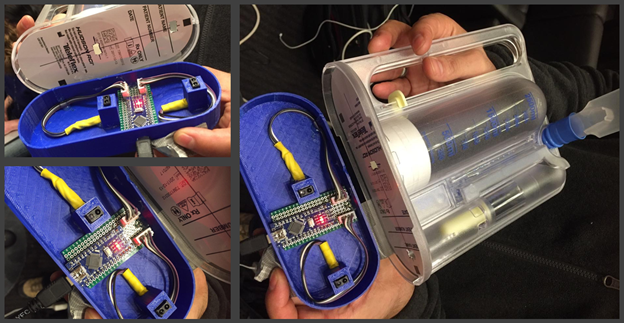


**Figure S2.** Pictures of the alternative incentive spirometer add-on device design consisting of two infrared reflective light sensors situated below the incentive spirometer and Arduino nano configuration. Raspberry Pi is wire-connected (not shown).

Sensor connectivity was changed between designs. Sensors were originally connected to a microcontroller by a solderless breadboard, utilizing an Arduino Nano (Arduno, A000005) and custom C++ code to interact with the sensors. Connections between components were made with breadboard jumper wires and resistors (Figure S2). The original design connected the Arduino Nano to a Raspberry Pi Zero W (Raspberry Pi), a small single-board computer which powered the device. In this original design, the Arduino reads the sensor data using custom C++ code and sends processed data to a connected laptop which was uploaded to the Raspberry Pi through a Python web application exposed by an Apache web server. Downstream applications were networked with the server through an API (application programming interface) to allow data collection and further utilities, such as video game integration.

Moving sensors out of the base provided space to incorporate additional add-on device improvements in data collection, data storage, and device power supply. The Arduino Nano was replaced with the ESP32 to decrease costs and incorporate Bluetooth communication. The original design communicated sensor data to a laptop computer through a Raspberry Pi via a web server, where the final design sent data directly to iPads via Bluetooth using the ESP32. With the original design, data could be collected only when an external application (ex. Apple iPad) was connected to the device. The final design incorporated a microSD card and reader for internal storage, allowing data collection independent of an external application. We were able to add a rechargeable lithium battery, charging module, and on/off switch. This allowed the device to be functional while unattached from a power source, allowing the device to become fully portable. With power on, the device now constantly reads data from the sensors and stores to the internal microSD card.
