# Supplementary material for: Development and Testing of a Data Capture Device for Use With Clinical Incentive Spirometers: Testing and Usability Study
Source: JMIR Biomed Eng. 2023 Sep 7;8:e46653. doi: 10.2196/46653 (PMC11041496; doi:10.2196/46653)
Supplement: Multimedia Appendix 4 [file biomedeng_v8i1e46653_app4.docx]

**Multimedia Appendix 4. Add-on device cost.**

**Table S1.** Hardware components purchased for ten add-on devices (estimates do not include tax, all values are in US dollars).

| **Description** | **Quantity** | **Unit Cost** | **Total Cost** |
| --- | --- | --- | --- |
| Side flow printed circuit board | 10 | $15.45 | $154.50 |
| Main volume printed circuit board | 10 | $15.32 | $153.20 |
| Base printed circuit board | 10 | $18.25 | $182.50 |
| 10K ohm resistors | 130 | $0.67 | $87.10 |
| 220 ohm resistors | 130 | $0.27 | $35.10 |
| 2N2222 transistors | 10 | $0.63 | $6.30 |
| ESP32 development boards | 10 | $28.23 | $282.30 |
| 3.7V 2AH lithium battery | 10 | $12.50 | $125.00 |
| Real time clock modules | 10 | $14.95 | $149.50 |
| Micro SD card reading module | 10 | $5.25 | $52.50 |
| SD card | 3 | $25.00 | $75.00 |
| Lithium battery charge module | 10 | $14.95 | $149.50 |
| Reflective optical sensors | 150 | $0.22 | $33.00 |
|  |  | **TOTAL** | **$1,485.50** |
